# Controlled suppression of the photoluminescence superlinear dependence on excitation density in quantum dots

**DOI:** 10.1186/1556-276X-7-551

**Published:** 2012-10-04

**Authors:** Sergio Bietti, Stefano Sanguinetti

**Affiliations:** 1 LNESS and Dipartimento di Scienza dei Materiali, Universitá di Milano-Bicocca, Via Cozzi 53, Milano, I-20125, Italy

**Keywords:** Quantum dots, Droplet epitaxy, Photoluminescence, Annealing

## Abstract

We have shown that it is possible to tune, up to complete suppression, the photoluminescence superlinear dependence on the excitation density in quantum dot samples at high temperatures by annealing treatments. The effect has been attributed to the reduction of the defectivity of the material induced by annealing.

## Background

Self-assembled semiconductor quantum dots (QDs) are zero-dimensional structures, being solid state systems with an atomic-like density of states. QDs are currently deeply investigated for their potentiality as building blocks for novel optoelectronic devices and for quantum information technologies. Several promising applications were recently developed in these fields, such as single photon sources for quantum cryptography [1], lasers [2,3], and optical amplifiers [4]. In all the foreseen applications, good knowledge of recombination kinetics and carrier dynamics at high temperature in the QDs is of the utmost importance.

Semiconductor QDs are known to efficiently capture the carriers generated by optical absorption. The electron hole pairs created in the barrier region rapidly relax to the QD ground state from where they radiatively recombine. The role of non-radiative channels on temperature dependence of the QD recombination efficiency has been analyzed in many details and with different experimental techniques [5-8]. It is nowadays well established that the high-T thermal quenching of the photoluminescence (PL) is due to the escape of carriers from the QD to the wetting layer or barrier material, where they undergo non-radiative recombination [9]. The presence of PL thermal quenching at intermediate temperatures has been attributed to the loss of carriers within the barriers during the relaxation path [9,10]. Despite the large experimental and theoretical effort devoted to the understanding of the carrier thermodynamics in QDs, a few aspects still need a better understanding.

In particular, it has been long debated, and still not completely clarified, whether the capture and escape of the carriers in QDs occur via single carrier [8,11,12] or electron-hole pairs [7,13-15]. In this discussion, a relevant point is taken by the interpretation of the superlinear dependence of the QD integrated PL (*I*_*L*_) on the excitation power density (*P*_exc_) at high T that is sometimes observed in QD samples (see, e.g., the works of Le Ru et al. and Sanguinetti et al. [8,14] and references therein). It is claimed that such observation is the fingerprint of a bimolecular recombination inside the QDs, thus inferring that single carrier dynamics dominates in the QDs [8,12]. On the contrary, on the basis of comparison of the temperature dependence of *I*_*L*_ on *P*_exc_ under non-resonant and resonant, with QD states, excitation conditions, Sanguinetti et al. [14] have attributed the superlinear *I*_*L *_behavior to the saturation of temperature-activated trap states, which affect the carrier diffusion in the barrier.

Here, we show that it is possible to turn off, in a controlled manner, the superlinear behavior of the QD *I*_*L*_ on *P*_exc_ at high T through the reduction of the non-radiative recombination centers of the QD material. This further supports the extrinsic origin of the observed PL superlinearity showing that it stems directly from a poor quality of the QD material. As the superlinearity is the outcome of temperature-activated non-radiative recombination centers in the barrier, we propose its suppression as an effective method to test the quality of the QDs and of the surrounding barrier.

For this study, we use QD samples fabricated by droplet epitaxy (DE) [16], which is a technique that allows for the fabrication of quantum nanostructures in lattice matched and mismatched systems with a precise control on the density, size, and shape. These achievements are due to the intrinsic high design flexibility of DE, making accessible a large variety of different structures, ranging from QDs [17], QD molecules [18], rings [19], multiple rings, and more complex shapes [20-22]. However, the DE growth is typically performed at low substrate temperature (between 150°C and 350°C). On one side, this allows to maintain a low thermal budget, making DE perfectly suited for the monolithic integration of GaAs QDs on CMOS devices [23]; on the other side, the crystalline quality of the grown materials at such low temperature is quite poor. Post-growth annealing is then commonly used to recover material quality. Different studies have been proposed to explain the effect of annealing on the QDs grown by DE, demonstrating the modifications induced on both morphological and electronic aspects of the nanostructure material [24-26].

## Methods

A single GaAs/AlGaAs QD sample is grown by DE in a conventional molecular beam epitaxy (MBE) system provided with an As-valved cell. After the growth of the Al_0.3_Ga_0.7_As barrier layer at 580°C by conventional MBE, the substrate temperature is lowered to 200°C and the As valve closed to deplete As pressure in the growth chamber. At this temperature and in the absence of As, only a flux of Ga atoms equivalent to 3.75 ML/s (where we indicate as 1 ML of Ga the amount of atoms necessary to obtain 1 monolayer of GaAs in the presence of As) is supplied at 0.5 ML/s rate. Due to the As-rich surface reconstruction, the initially deposited 1.75 ML/s are incorporated into the As terminated surface, resulting in the appearance of a Ga-stabilized surface, and the remaining 2 ML/s gives rise to the formation of tiny Ga droplets on the substrate. Following the deposition of the droplets, and maintaining the substrate temperature at 200°C, the As valve is opened to supply a molecular beam flux equivalent to 2 × 10^−4^ Torr beam on the surface. This growth step causes the complete arsenization of the Ga contained in the droplets, as demonstrated by the complete change of reflection high energy electron diffraction pattern from halo to spotty. A growth interruption of 10 min at 350°C under a constant As flux is then performed. This step did not introduce any change in the QD morphology [25]. The AFM images of the uncapped sample shows the formation of QDs, with an average height of ≈ 7 nm and a base of ≈ 30 nm. Finally, an Al_0.3_Ga_0.7_As barrier layer of 50 nm is grown by migration-enhanced epitaxy (MEE) [27] at 350°C. The sample is then cut in pieces and submitted to rapid thermal annealing (RTA) treatment in nitrogen atmosphere at different temperatures, namely 650°C (sample S650), 700°C (sample S700), 750°C (sample S750), 800°C (sample S800), and 850°C (sample S850). The annealing time is 4 min with a heating rate of 200°C/min. The PL spectra are measured between 15 and 210 K using a closed cycle cold finger cryostat. The PL is excited with an Nd:Yag duplicated laser (*λ*_exc_ = 532 nm). The excitation power density is 10 W/cm^2^. The spectra are measured by a grating monochromator operating with a Peltier cooled CCD camera.

## Results and discussion

### Results

**Figure 1:**
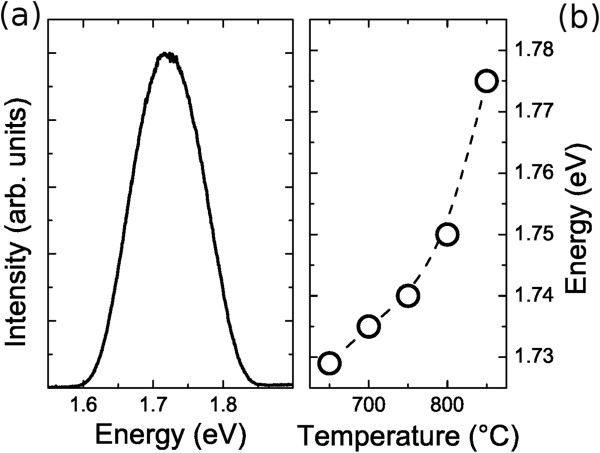
**PL spectrum and RTA temperature dependencies.** (**a**) PL spectrum of the S650 sample measured at *T *= 15 K. (**b**) PL spectrum centroid dependence on RTA temperature. The dashed line is a guide for the eyes.

The PL spectrum of the samples at *T *= 15 K show a broad emission band located between the GaAs and the Al_0.3_Ga_0.3_As energy gaps at 1.72 eV (see Figure 1a). With the increasing RTA temperature, a slightly blueshift is observed in QD-PL, as reported in Figure 1b. The amount of blueshift is in agreement with the model proposed by Sanguinetti et al. [28].

**Figure 2:**
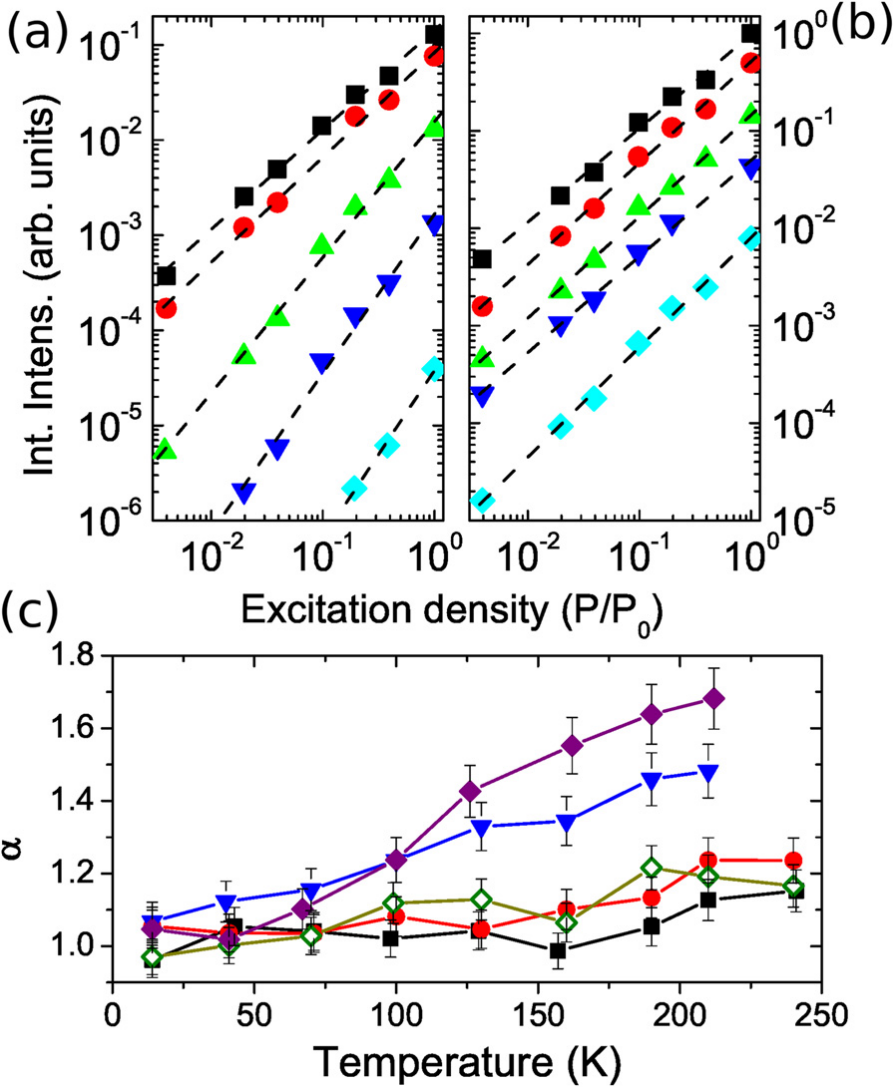
**PL excitation density dependence.** (**a, b**) Integrated intensities of the PL spectra versus excitation density at different temperatures for (a) sample S650 and (b) sample S850. Different symbols indicate the measurement temperatures, 15 K (squares), 70 K (circles), 130 K (upward triangles), 170 K (downward triangles), and 210 K (diamonds). The value of P_0_ is 70 W/cm^2^. The dashed lines are the fit of the experimental data with the equation $I_{L}=\beta P_{\text{exc}}^{\alpha }$. (**c**) Dependence of *α *on temperature for samples S650 (full diamonds), S700 (triangles), S750 (open diamonds), S800 (circles) and S850 (squares).

The dependence of the integrated PL intensity *I*_*L *_on the excitation density *P*_exc _was accurately measured over two decades at temperatures between 15 and 210 K. The data, for the samples S650 and S850, are reported in Figure 2a,b, respectively. The data are nicely fitted by a power law $I_{L}=\beta P_{\text{exc}}^{\alpha }$[14]. In Figure 2c, we report the values of temperature dependence of the the exponent *α* obtained from the fits of *I*_*L*_ for all the samples. At low temperature, the *I*_*L *_linearly depends on *P*_exc _in all the samples. Increasing the temperature, the samples with the highest RTA temperature continues to show a linear dependence of *I*_*L*_ on *P*_exc_. For the other samples, we observe a steady increase of *α*with the temperature. The slope of this rise monotonically increases as the annealing temperature of the sample decreases.

The increase of *α*with the temperature has been already observed in InAs/GaAs QDs and is attributed to the presence of a temperature-activated quenching channel [14] or to the transition of the carrier dynamics to the strong quenching regime [8]. What we observe here is that increase of *α *with the rising temperature can be reduced, an even eliminated, by a proper RTA treatment temperature. It is worth noting that the RTA treatment induces only minor changes in the electronic structure of the QD samples [26], as also shown by the small blueshift of the emission of sample S850. This rules out interpretation of the observed behavior as stemming from fundamental aspects of carrier dynamics.

**Figure 3:**
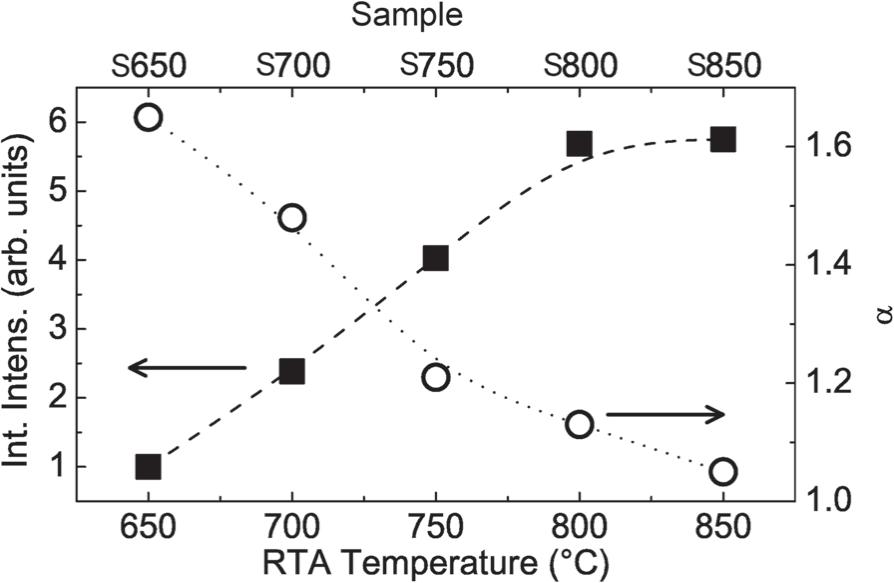
**PL spectrum and RTA temperature dependencies.** RTA temperature dependence of the integrated intensity (black squares) and of superlinear exponent *α*(open circles) of the QD PL spectra. The dotted and dashed lines are guides for the eyes.

The impact of the RTA treatment on the PL is clearly visible in Figure 3. The PL yield increases of a factor 6, raising the RTA temperature from 650°C to 800°C. Further increase of the annealing temperature (sample S850) does not appear to change the PL yield of the sample.

### Discussion

To understand this behavior, we have to recall the fact that fabrication of GaAs QDs by DE technique relies on maintaining a low substrate temperature and a high beam equivalent pressure As flux during the crystallization process, to prevent the migration of Ga atoms away from the initial Ga droplet. The crystallization at low temperature is accountable for the high defectivity and for possible under-stoichiometric composition of the as-grown material [28]. As a matter of fact, the low growth temperature of the DE QDs introduces a variety of lattice defects, including type III and type V vacancies, interstitials, and defect complexes. Aside from this step, it is also necessary to cover completely the GaAs QDs with a barrier layer grown at low temperature to protect the nanostructures and to freeze the achieved shape, preventing them from reaching the GaAs thermodynamical equilibrium state. To achieve good quality layers even at low growth temperature, MEE growth procedure is used [27]. Nevertheless, a defective AlGaAs layer in the region just close to the QDs is expected [26].

Increasing the substrate temperature after the deposition of a capping layer is expected to increase the mobility of the atoms, removing several defects without destroying the nanostructures. For this reason, post-growth thermal annealing is widely used to recover the poor crystalline quality of the as-grown DE QDs [24-26,28,29]. In fact, the RTA treatment induces interdiffusion of the group III species, which is promoted by the lattice defects introduced during the growth [28]. Disordered materials show increase of the interdiffusion of the Ga and Al species, promoted by group III vacancy-assisted diffusion. The interdiffusion of the group III species (in the order of few nanometers) reported during the post-growth annealing procedure is also responsible for the recovery of the crystalline quality of the QDs and of the barrier material. As reported in the work of Sanguinetti et al. [28], starting from out-of-equilibrium concentrations of vacancies presents in MDE materials, thermal annealing produces a depletion of the vacancy concentration due to surface evaporation or trap-driven decay of the excess, non-equilibrium, vacancies.

The observed dependence of the *α *temperature behavior on the RTA treatment temperature can be explained in terms of material defectivity and crystalline quality recovery. The superlinear dependence of integrated PL on the excitation power density (i.e., *α *> 1) implies that the internal quantum efficiency increases with raising the excitation power. This could be understood easily if we consider that the value of the efficiency is limited to 1. In the case of efficiency equal to 1, all the absorbed photons are re-emitted, so the ratio between the incoming and emitted photons is constant and the dependence between *I*_*L*_ and *P*_exc_ must be linear. On the other hand, in the case of samples where non-radiative recombination channels are present, the efficiency is reduced to values < 1. The increase of photogenerated carriers saturates the non-radiative recombination channels in the barrier, leading to a superlinear behavior and then *α *> 1. Therefore, the superlinearity of the QD emission intensity on the excitation power density could stem from the saturation of temperature-activated trap states, which affect the carrier diffusion in the barrier [14]. RTA treatment removes such defect, thus reducing the importance of this quenching channel, increasing the PL yield and progressively suppressing the *I*_*L*_ on *P*_exc _superlinerity. This can be appreciated in Figure 3 where the dependence of the PL yield and the *α* exponent at *T *= 190 K are reported. Clearly, *α *is in inverse relation with the PL yield, thus supporting the interpretation of the PL superlinarity as stemming from defect-related effects.

This has also an interesting applicative counterpart. The measurement of the *α *exponent at high temperatures makes it possible to evaluate the quality of the QDs and of the surrounding barrier material by simple PL measurements. A linear dependence of *I*_*L*_ on *P*_exc _demonstrates the absence of temperature-activated non-radiative decay in the material.

## Conclusions

In conclusion, we have shown that it is possible to tune, up to complete suppression, the PL superlinear dependence on the excitation density in QD samples at high temperatures by RTA treatments. The effect has been attributed to the reduction of the defectivity of the material induced by the annealing. This rules out possible explanation of the superlinear behavior as stemming from intrinsic carrier dynamics effects.

## Competing interests

Both authors declare that they have no competing interests.

## Authors’ contributions

SS proposed and guided the overall project. SB performed material growth, AFM and PL measurements. Both authors read and approved the final manuscript.
